# Toxicity of binary mixtures of Cu, Cr and As to the earthworm *Eisenia andrei*

**DOI:** 10.1007/s10646-020-02240-1

**Published:** 2020-06-25

**Authors:** Johanna Kilpi-Koski, Olli-Pekka Penttinen, Ari O. Väisänen, Cornelis A. M. van Gestel

**Affiliations:** 1 Department of Environmental Sciences, Faculty of Biological and Environmental Sciences, University of Helsinki, Niemenkatu 73, 15140 Lahti, Finland; 2 Department of Chemistry, University of Jyväskylä, PL 35, 40014 University of Jyväskylä, Finland; 3 Department of Ecological Science, Faculty of Science, Vrije Universiteit, De Boelelaan 1085, 1081 HV Amsterdam, The Netherlands

**Keywords:** Mixture toxicity, CCA metals, Bioavailability, MIXTOX model, Freundlich sorption isotherms

## Abstract

Chromated copper arsenate (CCA) mixtures were used in the past for wood preservation, leading to large scale soil contamination. This study aimed at contributing to the risk assessment of CCA-contaminated soils by assessing the toxicity of binary mixtures of copper, chromium and arsenic to the earthworm *Eisenia andrei* in OECD artificial soil. Mixture effects were related to reference models of Concentration Addition (CA) and Independent Action (IA) using the MIXTOX model, with effects being related to total and available (H_2_O and 0.01 M CaCl_2_ extractable) concentrations in the soil. Since only in mixtures with arsenic dose-related mortality occurred (LC_50_ 92.5 mg/kg dry soil), it was not possible to analyze the mixture effects on earthworm survival with the MIXTOX model. EC_50_s for effects of Cu, Cr and As on earthworm reproduction, based on total soil concentrations, were 154, 449 and 9.1 mg/kg dry soil, respectively. Effects of mixtures were mainly antagonistic when related to the CA model but additive related to the IA model. This was the case when mixture effects were based on total and H_2_O-extractable concentrations; when based on CaCl_2_-extractable concentrations effects mainly were additive related to the CA model except for the Cr–As mixture which acted antagonistically. These results suggest that the CCA components do interact leading to a reduced toxicity when present in a mixture.

## Introduction

Following over 200 years of industrialization, soil contamination is a widespread problem in many countries. According to data collected through a European Network, mineral oil and metals are the main contaminants contributing 60% to soil contamination in Europe (Panagos et al. 2013). Especially metals and persistent organic compounds can still be found at high concentrations decades after the emissions have ceased (Hagner et al. 2017). Metals enter the environment through different anthropogenic sources like mining, traffic, smelters and the emission of combustion by-products.

Finland has around 24,000 contaminated sites, including 200 sites used for wood salt impregnation (Pyy et al. 2013) polluted by the inorganic wood impregnation chemical chromated copper arsenate (CCA) posing a high environmental risk (Karjalainen et al. 2009). The need for a risk assessment of CCA-contaminated soils is evident because contaminants have complex relationships with the natural hydrogeochemical environment and biota. For example, the reduced abundance of decomposer organisms and the effects observed in invertebrate toxicity tests imply that risks to the soil ecosystem do occur. However, one of the major conclusions of an earlier study was that to accurately assess the interactions of the metals in CCA-contaminated soils and to determine the metal(s) most contributing to the actual risk at wood treatment sites, more tests are needed both on the single metals and their mixtures (Karjalainen et al. 2009).

Some studies dealt with the distribution and mobility of the CCA metals in soil (Balasoiu et al. 2001), and the toxicity to soil invertebrates of the single metals is generally well known (Peijnenburg et al. 1999b; Spurgeon and Hopkin 1996; Leduc et al. 2008; Meharg et al. 1998; Nahmani et al. 2007). The complexity and the challenges of metal toxicity in soil have recently been summarized by Moyson et al. (2018) who stated that the effects of metal mixtures are not yet well understood and that there is a notable paucity of information on their effects on soil invertebrates. The interaction effects are dependent on the metal combinations employed (Moyson et al. 2018). Interactions may occur at different steps of the intoxication process, like sorption to the soil determining metal availability, the uptake in the organism determining body concentrations and the interaction with the target site determining toxicity (Van Gestel and Hensbergen 1997). Therefore, information regarding interaction patterns of the CCA metals is highly relevant. Such interactions, however, have not been investigated so far.

In earthworms, Cu is mainly regulated by the metallothionein protein (Fisker et al. 2011). Chromium and copper showed similar fast uptake and elimination patterns in the earthworm *Eisenia andrei*, while arsenic uptake and elimination was slow, not reaching steady state within 3 weeks (Kilpi-Koski et al. 2019). These differences in uptake kinetics cause that effects of exposure to mixtures of the CCA metals are hard to predict. To assess the potential risk of metal pollution to terrestrial ecosystems, mixture toxicity tests are needed because the single metals may interact, potentially leading to higher or lower toxicity of the mixture than expected (Van Gestel et al. 2011).

In this present study we exposed the earthworm *Eisenia andrei*, as a representative of an important and ecologically relevant group of soil invertebrates, to binary mixtures of Cu, Cr and As. We determined deviations of mixture toxicity from the reference models of concentration addition (CA) and independent action (IA) (Gomez-Eyles et al. 2009; Van Gestel et al. 2011) by applying the MIXTOX model of Jonker et al. (2005). Toxicity tests were performed simultaneously with the individual metals and the binary mixtures at various concentration ratios. Mixture effects were compared to effects of the single metal, and related to total and available concentrations in the soil.

Our hypotheses were that As, Cr and Cu in binary mixtures would be more toxic than the single metals alone in affecting earthworm survival and reproduction. This assumption of synergistic interactions between the CCA metals is based on the dissimilarity in their modes of action and the differences in toxicokinetics in *E. andrei* found in our earlier study (Kilpi-Koski et al. 2019).

## Materials and methods

### Earthworms

Earthworms *Eisenia andrei* have been cultured at the Vrije Universiteit, Amsterdam, The Netherlands for many years. The earthworm cultures were fed with horse dung, free of pharmaceuticals or any other contaminants. For the tests, only adult earthworms with a well-developed clitellum were used. Before use in the tests, the earthworms were acclimatized in OECD artificial soil (OECD 1984) for 24 h at 20 ± 1 °C and a light:dark cycle of 16/8 h.

### Preparation of the test soils

The earthworms were exposed in OECD artificial soil (OECD 1984). The artificial soil contained 10% <2 mm sphagnum peat, 70% <1 mm quartz sand and 20% kaolin clay and 0.4% CaCO_3_, on a dry weight basis, which were thoroughly mixed using a household mixer. After mixing, pH-H_2_O was 6.27 and pH-CaCl_2_ 5.79. Soil moisture content was brought to 71% (w/w), which corresponds with 50% of the water holding capacity (WHC).

Stock solutions of K_2_CrO_4_ (Sigma-Aldrich ≥99.0%), CuCl_2_*2H_2_O (Sigma-Aldrich ≥99.0%) and Na2HAsO4*7H_2_O (Sigma-Aldrich ≥99.0%) in water were used to spike the artificial soil. In this way, water content of the soil was adjusted to the right level when introducing the metals.

Three binary mixtures (copper-arsenic, copper-chromium and chromium-arsenic) were studied. The experimental design for the binary mixture experiment was based on the toxic unit (TU) concept with reproduction as the endpoint, with TU defined as:

$$TU = \frac{c}{{EC50}},$$

where EC_50_ is the median effective concentration causing 50% reduction of earthworm reproduction (in mg/kg dry soil), and c is the metal concentration in the mixture (in mg/kg dry soil).

Test concentrations chosen were based on the few available data on the toxicity of the three metals (Cu, Cr and As) to earthworms (Koster et al. 2006; Sivakumar and Subbhuraam 2005; Langdon et al. 2001a).

The mixtures tested had metal ratios of 1:1, 9:1, 1:9, 1:3 and 3:1. See Fig. S1 in the Supporting Information for the experimental design. After introduction of the metals, the artificial soil was equilibrated for three weeks before starting earthworm exposures.

### Toxicity testing

Portions equivalent with 500 g dry weight artificial soil were put into 800 ml glass jars. Considering the work load of a full mixture toxicity test and the fact that a regression-based model was used for data analysis, only three replicates (instead of four) were used for each concentration and five controls were included. At the start of the experiments, 9 earthworms (*E. andrei*) were taken from the acclimatization, rinsed in water, blotted dry on filter paper, weighed and added into each jar, together with 2 g of horse dung for food. Because of the lack of sufficient adult earthworms, tests were performed with 9 instead of 10 earthworms per test jar. The jars were loosely closed with a lid and incubated in a climate room at 20 ± 0.1 °C, 75% relative humidity and a 8/16 h light/dark cycle. Soil moisture content was checked once a week by weighing the jars and replenishing the water loss with deionized water. Additional horse dung was added if needed. After 4 weeks of incubation, survival and mass of the surviving earthworms were determined. Soil without earthworms was returned into the jars and incubated for another 4 weeks. After this period, the number of juveniles produced was determined by placing the jars in a water bath at 60 °C. Juveniles emerging to the surface were collected and counted.

### Chemical analysis

About 500 mg (dw) artificial soil was weighed into 50 ml plastic bottles and 10 ml aqua regia (3:1 HCl:HNO_3_) was added. The acids (HCl 36.5–38.0% and HNO_3_ 69.0–70.0%) were supplied by J.T. Baker for trace metal analysis. The closed bottles were placed in an ultrasonic bath (Transsonic 820/H Elma^®^) for 3 × 3 min sonification at 45–50 °C. After sonification and cooling the samples were filtered (Whatman No 41.), diluted with high purity ELGA-water to a volume of 25 ml, and stored in plastic bottles for analysis with Inductively Coupled Plasma Optical Emission Spectrometry ICP-OES (Perkin Elmer Optima 4300DV). All the equipment was rinsed with acid before use. Reference materials SRM 2710 and SRM 2711, both certified by the National Institute of Standards and Technology (NIST), were included to check for the quality of the soil analysis. Recovery from certified reference sample SRM 2710 was 96% for As and 92% for Cu, and from sample SRM 2711 it was 96% for Cu. Unfortunately, no certified reference sample was available for Cr, but the high recoveries for Cu and As suggest that the spiking method used was appropriate in achieving the desired soil concentrations. The procedure has been described in more detail by Väisänen et al. (2002).

To determine water-extractable and CaCl_2_-exchangeable metal concentrations, about 5 g moist soil was extracted with 50 ml H_2_O or 50 ml 0.01 M CaCl_2_, respectively by shaking for 2 h at 200 rpm. After settling overnight, pH was measured, and the overlaying solutions were 0.45 µm filtered and conserved with HNO_3_ for analyzing extractable metal concentrations (Smit et al. 1997). Extractable metal concentrations were analyzed by ICP-OES (Perkin Elmer Optima 4300DV).

## Model and statistics

### Metal partitioning in soil

To assess metal partitioning in the soils, Freundlich sorption isotherms were fitted to the measured total and extractable concentrations: Eq. 1.

1 $$C_s = K_f \times C_{ext}^n,$$

where C_s_ is total concentration in soil (mg/kg dry soil), C_ext_ the concentration in the H_2_O or CaCl_2_ extract (mg/L), K_f_ the Freundlich sorption constant ((L/kg)^n^), and n indicates the deviation from linearity. K_f_ and n were estimated from linear plots of log C_s_ versus log C_ext_, in which control values were omitted.

### Toxicity of single metals

Single metal toxicity data were fitted to logistic and, if applicable, hormetic dose–response models.

The logistic model for estimating EC_50_ and EC_10_ is given in Eqs. 2 and 3.

2 $$Y = \frac{{Y_{max}}}{{{\mathrm{1}} + \left( {\frac{c}{{EC_{50}}}} \right)^{slope}}},$$

3 $$Y = \frac{{Y_{max}}}{{1 + \left( {\frac{{10}}{{90}}} \right) \times \left( {\frac{c}{{EC_{10}}}} \right)^{slope}}}.$$

The hormesis model (Van Ewijk and Hoekstra 1993) is presented in Eqs. 4 and 5 for EC_50_ and EC_10_ estimates respectively.

4 $$Y = \frac{{Y_{max}(1 + f \times c)}}{{1 + \left( 2f \times EC_{50} + 1 \right) \times \left( {\frac{c}{{EC_{50}}}} \right)^{slope}}},$$

5 $$Y = \frac{{Y_{max}(1 + f \times c)}}{{1 + \left( {\frac{{10}}{{90}}} \right) \times \left(2f \times EC_{10} + 1 \right) \times \left( {\frac{c}{{EC_{10}}}} \right)^{slope}}}.$$

In these equations, Y_max_ is the maximum response, c the exposure concentration, EC_50_ and EC_10_ the concentrations reducing the response by 50% and 10% compared to the control, f the hormesis parameter, and the slope indicates the steepness of the dose–response curve. Values for these parameters and corresponding 95% confidence intervals were obtained by using the nonlinear fitting procedure in SPSS.

### Mixture toxicity

Mixture toxicity data were analyzed using the MIXTOX model developed by Jonker et al. (2005). This model allows for comparing observed data with mixture effects expected using the concentration addition (CA) and the independent action (IA) reference models. The model used the effects seen in the single metal exposures, which were run simultaneously with each mixture experiment (see Fig. S1), as the starting point. The model was applied for every binary mixture and for every metal pool (measured total, and water or CaCl_2_ extractable concentrations) to assess mixture effects on the reproduction of *E. andrei*. It was first tested for possible deviations from the reference model. If deviations were seen, the CA and IA models were extended with deviation functions including extra parameters to describe synergistic/antagonistic, dose-level and dose-ratio dependency (Loureiro et al. 2010). Data were fitted to the model using the solver function in Microsoft EXCEL.

## Results

### Metal availability

Soil pH-H_2_O was 6.21–6.38 and pH-CaCl_2_ 5.75–5.83. Actual metal concentrations were 69–81%, 74–82% and 90–97% of the nominal ones for Cu, Cr and As, respectively (Table 1). H_2_O- and 0.01 M CaCl_2_-extractable concentrations were similar and increased with increasing total soil concentration (Table 1). H_2_O-extractable concentrations were 5.0–18.5% of the total measured concentrations for Cu, 4.8–10.9% for Cr and 47.2–115% for As. Corresponding CaCl_2_-extractable concentrations were 5.14–13.0%, 2.37–9.0% and 62.4–95.6% of the total concentration for Cu, Cr and As, respectively.

**Table 1:** Nominal and measured total and H_2_O- and 0.01 M CaCl_2_-extractable Cu, Cr and As concentrations (mg/kg dry soil) of the single treatments in OECD artificial soil, determined at the beginning of the mixture toxicity experiments with *Eisenia andrei*. All measured concentrations are mean values ± standard deviation (*n* = 4)

Total Cu			
Nominal	Actual	H_2_O-extractable	CaCl_2_-extractable
0	0.40 ± 0.28	0.27 ± 0.00075	0.24 ± 0.002
50	38.1 ± 3.52	1.90 ± 0.08	1.96 ± 1.11
100	74.0 ± 3.10	4.07 ± 0.37	3.69 ± 0.23
200	161 ± 2.86	9.92 ± 1.01	8.67 ± 1.12
400	324 ± 48.0	25.6 ± 2.93	22.9 ± 1.44
800	557 ± 194	102 ± 4.79	72.5 ± 39.6
Total Cr			
0	5.07 ± 3.53	3.90 ± 5.18	0.12 ± 0.004
38.4	30.0 ± 1.91	3.28 ± 0.32	2.70 ± 0.47
96	78.4 ± 7.07	7.82 ± 0.68	6.66 ± 0.65
240	196 ± 12.6	14.0 ± 1.68	12.4 ± 1.39
600	445 ± 52.0	31.2 ± 7.98	26.5 ± 7.37
1500	1224 ± 51.0	59.3 ± 0.50	44.2 ± 10.6
Total As			
0	2.18 ± 0.81	1.03 ± 0.02	1.36 ± 0.05
15.4	14.6 ± 2.26	12.3 ± 1.42	12.1 ± 1.06
38.4	33.5 ± 4.86	33.9 ± 3.13	32.0 ± 2.37
96	86.9 ± 11.7	100 ± 58.3	78.9 ± 13.9
240	232 ± 90.3	194 ± 16.1	177 ± 56.3
600	570 ± 175	529 ± 74.2	530 ± 58.5

All measured concentrations are mean values ± standard deviation (*n* = 4)

### Partitioning/sorption of the metals

Tables S1–S3 (in the Supporting Information) show the Freundlich parameters for the sorption of the three metals, single and in the binary mixtures; Figs. S2–S4 compare the Freundlich K_f_ values for the different metal combinations. Compared to the single Cu treatment, the sorption of Cu was slightly lower at the lowest Cu:metal ratio, but higher for all other mixtures with As and Cr (Fig. S2). In the binary mixtures with Cr, there was strange outlier at Cr–Cu 50:50 but the overall trend was again a lower sorption at the 10:90 Cr–Cu ratio and increased sorption at the high Cr–Me ratios (Fig. S3). The interactions in the binary mixtures with As were more scattered, with a stronger sorption at low As-Me ratio but a lower sorption at the 30:70 As–Cr ratio that increased with increasing ratio of As–Cr (Fig. S4). Such trend was not visible for Cu.

### Metal toxicity

**Fig. 1:**
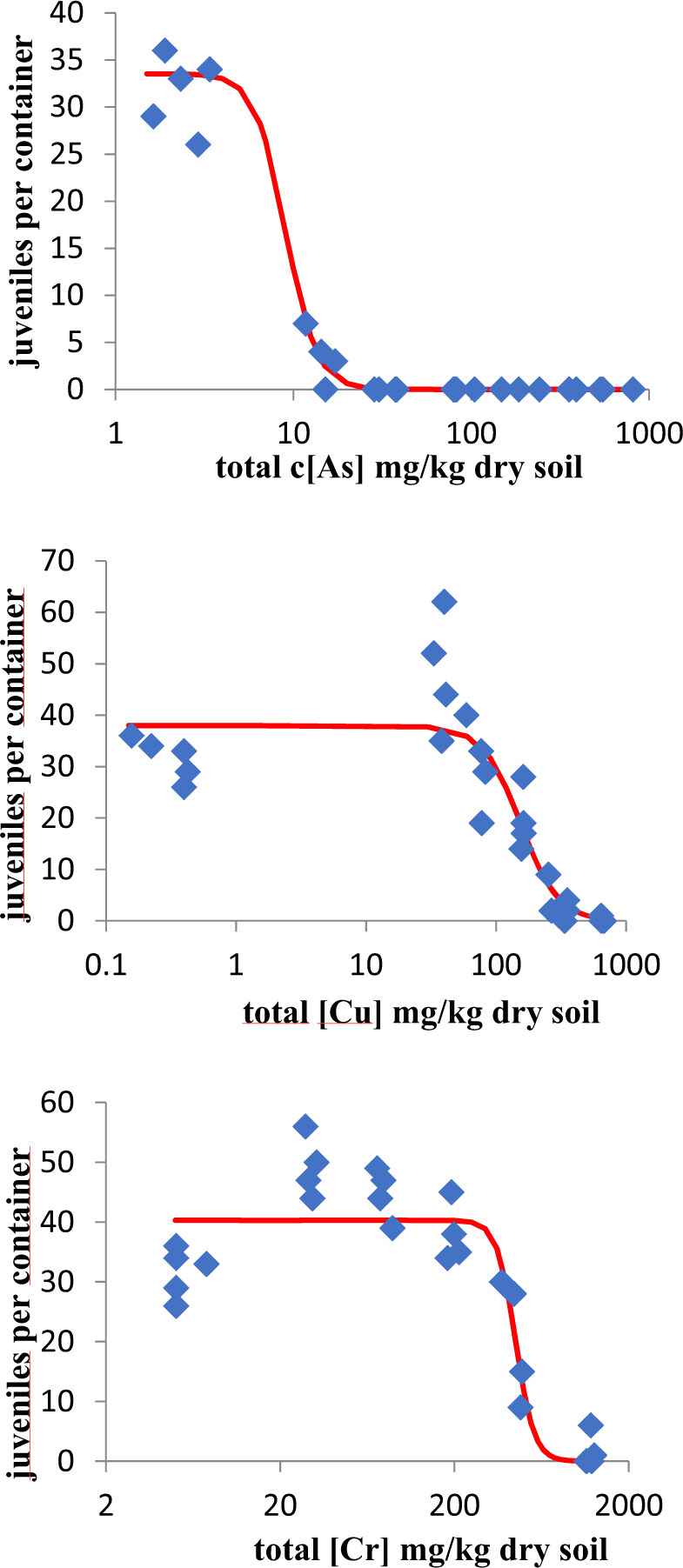
Effects of the single metals As, Cu and Cr on the reproduction of *Eisenia andrei* after 8 weeks exposure in OECD artificial soils. Lines show the fit to the data of a logistic dose–response model; R^2^ values for the goodness of fit of the curves were 0.981, 0.793 and 0.824 for As, Cu and Cr, respectively. See Table 2 for the EC_50_ and EC_10_ values calculated from the dose–response curves

Control survival of the earthworms was 100%, while the number of juveniles produced in the controls was 31.6 ± 4.04 (±SD, *n* = 5) and coefficient of variation was 12.8%. Cu and Cr did not affect earthworm survival at the concentrations tested, but As caused a dose-related increase of mortality with an estimated LC_50_ of 92.5 (70.1–122) mg As/kg dry soil. The single metal toxicity data for effects on earthworm reproduction were reasonably well fitted using the logistic model (Eq. 2) for all three metals (Fig. 1). Hormetic effects were found at low concentrations of Cr and Cu, these data were therefore also fitted using Eq. 4 (Fig. S5). The AIC values suggested a slightly better fit of the hormetic dose–response model to the Cu and Cr data than the logistic model. Both models gave similar EC_50_ values, but EC_10_s estimated with the hormesis model were much lower (Table 2). EC_50_s calculated with the hormesis model for Cr and Cu were 546 and 148 mg/kg in dry soil, respectively. Since the hormetic response might also be due to the fairly low control performance, for the mixture toxicity analysis the logistic model was used.

**Table 2:** EC_50_ and EC_10_ values (in mg/kg dry soil) for the effects of Cu, Cr and As on the reproduction of *Eisenia andrei* in OECD artificial soil (see Fig. 1 for corresponding dose–response curves)

	Cu		Cr		As	
Expression of exposure	EC_50_	EC_10_	EC_50_	EC_10_	EC_50_	EC_10_
Total concentration—logistic model	154 (106–202)	74.0 (15.1–133)	449 (399–499)	343 (215–472)	9.08 (5.96–12.2)	5.82 (1.41–10.2)
Total soil concentration—hormesis model	148 (101–194)	28.1 (5.74–50.5)	546 (279–813)	67.4 (29.0–106)	N.A.	N.A.
H_2_O extractable	9.70 (5.62–13.8)	3.66 (−0.01 to 7.43)	26.8 (24.6–29.0)	23.7 (17.6–29.4)	5.76 (−1.60 to 13.1)	2.66 (−4.26 to 9.57)
CaCl_2_ extractable	9.01 (5.51–12.5)	3.92 (0.39–7.45)	23.9 (16.6–31.2)	11.9 (3.62–20.1)	5.96 (−3.76 to 15.7)	2.95 (−6.59 to 12.5)

In between brackets, the corresponding 95% confidence intervals are given. All values are estimated using a logistic dose–response model. For Cu and Cr, also EC50 and EC10 values based on measured total soil concentrations are included that were estimated using a hormesis model (see Fig. S4 in the Supporting Information)

*N.A.* not applicable

### Binary mixture exposures

Only in the mixtures with arsenic, significant and dose-related mortality occurred. Since no mortality occurred in the single Cr and Cu exposures, it was not possible to analyze the mixture effects on earthworm survival using the MIXTOX model. A likelihood ratio test showed that the interaction of Cu and As was significantly antagonistic (X^2^_(df=1)_ = 5.15, p < 0.05) while that of Cr and As was not (X^2^_(df=1)_ = 1.97, n.s.).

Results of the analysis of the effects of the binary metal mixtures of Cu–As, Cu–Cr and Cr–As on the reproduction of *E. andrei* using the reference models of concentration addition (CA) and independent action (IA) are shown Tables 3–5 when related to total metal concentrations. In the Supporting Information Tables S4–S6 and S7–S9 show mixture effects based on H_2_O and 0.01 M CaCl_2_ extractable concentrations, respectively. The results of all mixture toxicity calculations are summarized in Table 6.

**Table 3:** Effects of a binary mixture of copper (Cu)–arsenic (As) on the reproduction of *Eisenia andrei* (number of juveniles per pot) exposed for 8 weeks in spiked OECD artificial soil, based on measured total soil concentrations. Values printed in bold indicate the best model fit and were used for drawing conclusions on the toxicity of the mixture

	Total							
			S/A		DR		DL	
	CA	IA	CA	IA	CA	IA	CA	IA
Max	35.6	36.5	36.0	36.7	35.9	36.8	35.5	36.0
Slope Cu	4.95	2.95	3.51	2.47	3.74	2.25	With slope fixed at 3.75	2.47
Slope As	7.46	6.97	7.98	6.05	7.50	6.16	7.50	2.45
EC_50_ Cu (mg/kg)	274	154	158	103	189	126	164	104
EC_50_ As (mg/kg)	13.3	11.7	11.2	10.5	11.0	10.3	11.2	6.59
*a*			**1.96**	3.02	2.40	5.0	2.69	30.4
*b*					−1.72	−5.69	0.20	0.97
chi = p(*x*^2^)			**3.1** **×** **10**^**−**^^**5**^	0.17	0.408	0.41	0.70	0.17
*R*^2^	0.803	0.831	0.836	0.835	0.837	0.836	0.836	0.838

Values are calculated using the concentration addition model (CA) and the independent action model (IA) extended with deviation parameter *a* to show synergism/antagonism (S/A) and an additional deviation parameter *b* for dose ratio-dependent (DR) or dose level-dependent deviations (DL). The p(*X*^2^) values indicate the significance of the additional deviation parameters

**Table 4:** Effects of a binary mixture of copper (Cu)–chromium (Cr) on the reproduction of *Eisenia andrei* (number of juveniles per pot) exposed for 8 weeks in spiked OECD soil based on total soil concentrations. Values printed in bold indicate the best model fit and were used for drawing conclusions on the toxicity of the mixture

	Total							
			S/A		DR		DL	
	CA	IA	CA	IA	CA	IA	CA	IA
Max	41.3	40.4	40.5	40.5	39.7	40.4	40.4	41.2
Slope Cu	2.40	2.50	2.38	2.47	2.39	2.52	2.36	2.52
Slope Cr	2.04	2.68	3.16	2.44	7.37	2.54	3.16	2.25
EC_50_ Cu (mg/kg)	141	128	117	120	134	132	117	116
EC_50_ Cr (mg/kg)	596	544	495	517	450	479	496	493
*a*			**2.06**	0.96	**4.15**	4.87	2.21	0.002
*b*					**−4.21**	−7.27	0.04	−1290
chi = p(*x*^2^)			**0.0021**	0.37	**0.0436**	0.16	0.914	0.41
*R*^2^	0.764	0.782	0.786	0.784	0.795	0.790	0.786	0.786

Values are calculated based on the concentration addition model (CA) and the independent action model (IA) extended with deviation parameter *a* is showing synergism/antagonism (S/A) and an additional deviation parameter *b* for dose ratio-dependent (DR) or dose level-dependent deviations (DL). The p(*X*^2^) values indicate the significance of the additional deviation parameters

**Table 5:** Effects of a binary mixture of chromium (Cr)–arsenic (As) on the reproduction of *Eisenia andrei* (number of juveniles per pot) exposed for 8 weeks in spiked OECD soil based on total concentrations. Values printed in bold indicate the best model fit and were used for drawing conclusions on the toxicity of the mixture

	Total							
			S/A		DR		DL	
	CA	IA	CA	IA	CA	IA	CA	IA
Max	37.1	37.6	37.9	36.8	37.9	36.9	40.2	35.0
Slope Cr	2.27	10.5	1.54	Fixed at 2.5	1.23	Fixed at 2.5^a^	Fixed at 1.23	Fixed at 10
Slope As	6.08	6.09	4.92	3.76	Fixed at 7.5	4.10	Fixed at 7.5	7.38
EC_**50**_ Cr (mg/kg)	1170	460	206	219	396	434^a^	202	270
EC_50_ As (mg/kg)	11.2	10.5	5.98	6.8	5.77	7.09	6.24	5.80
*a*			**8.10**	**11.8**	**14.1**	**21.1**	5.64	316
*b*					**−16.2**	**−26.2**	−0.077	0.92
chi = p(*x*^2^)			**1.6** × **10**^**−9**^	**0.0002**	**0.041**	**0.0058**	0.14	0.061
*R*^2^	0.739	0.778	0.819	0.807	0.826	0.821	0.823	0.814

Values are calculated based on the concentration addition model (CA) and the independent action model (IA) extended with deviation parameter *a* is showing synergism/antagonism (S/A) and an additional deviation parameter b for dose ratio-dependent (DR) or dose level-dependent deviations (DL). The p(*X*^2^) values indicate the significance of the additional deviation parameters

^a^IA model with slope of Cr fixed at 2.5 very unrealistic values for EC_50_

**Table 6:** Summary of the results on the toxicity of binary mixtures of As, Cr and Cu to the reproduction of *Eisenia andrei* in OECD artificial soil, based on total and extractable concentrations (H_2_O and 0.01 M CaCl_2_)

Mixture	Expression of exposure	CA	IA
Cu–As	Total	Antagonism	Additivity
	H_2_O extract	Antagonism	Additivity
	CaCl_2_ extract	Additivity	Synergism for S/A, no further DR and DL deviations
Cu–Cr	Total	Antagonism	Additivity
	H_2_O extract	Antagonism; DR shows that the mixture is more antagonistic at increasing Cu concentration	Additive, but tendency to DR with antagonism at increasing Cu concentration
	CaCl_2_ extract	Additivity	Synergism for S/A, no further DR and DL deviations
Cr–As	Total	Antagonism; DR, antagonistic at increasing Cr concentration	Antagonism; DR, antagonistic increasing Cr concentration
	H_2_O extract	Antagonism	Antagonism
	CaCl_2_ extract	Antagonism	Antagonism

Results are shown from the analyses using the concentration addition (CA) and independent action (IA) model. See Tables 3–5 and S4–S9 for the individual mixture responses

For the Cu–As mixture, there was overall antagonism when tested against the CA model (*a* = 1.96), without further deviations. The data did fit the Independent Action model without any further deviations. The same pattern was seen when mixture effects were related to H_2_O-extractable concentrations. When related to 0.01 M CaCl_2_-extractable concentrations, mixture effects, however, were additive when analyzed using the CA model and synergistic according to the IA model (Table 3, S4 and S7).

For the Cr–Cu mixture, there was overall antagonism when tested against the CA model, with significant DR dependent deviations. The values of *a* = 4.15 and *b* = −4.21 indicate synergism when copper dominates the mixture and antagonism when chromium is the dominating element. Interactions were best explained from the Independent Action model. When related to H_2_O-extractable concentrations, the Cr–Cu mixture acted antagonistic according to the CA model and additive according to the IA model, with in both cases a dose-related dependency suggesting a switch from antagonism to synergism already at concentrations far below the EC_50_. When based on 0.01 M CaCl_2_-extractable concentrations, the Cr–Cu mixture acted additively according to the CA model and synergistic according to the IA model (Table 4, S5 and S8).

For the Cr–As mixture, both the CA and the IA model indicated antagonism. The CA model showed slight DR or DL dependent deviations, which were significant only when unrealistically large slopes of the dose–response curves were allowed. These deviations therefore were not considered realistic. The IA model pointed into the direction of DR-dependent deviations; *a* = 21.1 (fixed slope) means antagonism, expect for those mixture ratios where significant negative b values indicate synergism. The parameter value *b* = −26.2 (fixed slope) suggests that synergism is mainly seen when As is dominating the mixture. When related to H_2_O and CaCl_2_-extractable concentrations, the Cr–As mixture acted antagonistic according to both the CA and IA models (Table 5, S6 and S9).

## Discussion

In this study, similar metal extractabilities were found for H_2_O and 0.01 M CaCl_2_. Freundlich parameters in some cases were influenced by metal/metal ratios. The binary mixture effects were overall antagonistic when related to the CA model and additive when related to the IA model. Deviations from this pattern were sometimes seen when relating effects to extractable concentrations.

### Metal partitioning

H_2_O- and 0.01 M CaCl_2_-extractable concentrations were similar and increased with increasing total soil concentration. For cationic metals, like Cu, 0.01 M CaCl_2_-extractable concentrations generally are higher than H_2_O extractable concentrations because of cation exchange effects (Giska et al. 2014; Hobbelen et al. 2006). Sauvé et al. (1997) however, also found higher H_2_O than CaCl_2_ extractable Cu concentrations in urban and agricultural soils with pH(CaCl_2_) 6.99–7.62 and 6.03–7.28 and OM contents of 0.41–10.77% and 1.57–6.35%, respectively. Ca may have promoted coagulation of soluble organic matter (Sauvé et al. 1997) reducing the mobility of Cu by complexation with fulvic and humic acids (McLaren et al. 1981). Giska et al. (2014) found higher 0.01 M CaCl_2_ extractable Cu fractions (0.04–0.14%) than H_2_O extractable fractions (0.12–0.44%) in field-contaminated soils with a high OM content (36.3–54.2%) but lower pH-CaCl_2_ (3.46–5.06) and total Cu concentrations of 27.2–67.0 mg/kg (dw). And Hobbelen et al. (2006), using field-contaminated soils with high pH-CaCl_2_ (>7), OM content (15.1–30.0%) and clay content (14.7–46.3%), reported CaCl_2_-extractable Cu fractions of 0.02–0.20% from the total concentrations. These extractable fractions in field soils are lower than the ones in our test (5.0–18.5%), which might be due to the lack of ageing and the use of a freshly spiked artificial soil which has a lower sorption capacity for metals.

The pH in our soil was in the range that may influence precipitation of Cu, Cr and As, making it likely they formed complexes (Kim et al. 2015; Langdon et al. 2002; Mesuere and Fish 1992). Schultz et al. (2004) found that the majority of As and Cr was less mobile because of the formation of complexes that firmly bound to clay minerals. The complexes formed may bind to soil particles (Balasoiu et al. 2001) which will have affected metal availability. Balasoiu et al. (2001) studied the fate of a commercial CCA-C-solution containing 45.5% CrO_3_, 18.2% CuO and 36.3% As_2_O_5_ in artificial soils with different combinations of kaolinite (5–30%), sand (30–69.5%), organic matter (0.5–15%) and 25% silt. The measured pH (H_2_O) was in the same range as in our study. Metal retention in the mineral artificial soil was low at 58% for Cu and at 23% for Cr but increased in high organic artificial soils to 96% for Cu and 78% for Cr. Cr is anionic metal, but its speciation form Cr(III) exists as cationic species at pH < 4 (Reijonen 2017*)*. In soils, the mobile Cr(VI) species will be converted into the more stable Cr(III) (Kumpiene et al. 2008). Cr(III) adsorption is influenced by pH and cation exchange capacity (Choppala et al. 2010). Cr(III) was adsorbed very strongly at pH < 4 by both kaolinite and montmorillonite indicating a low mobility in soils. At pH 4–5, a combination of adsorption and precipitation processes made Cr(III) quite immobile in soil (Griffin et al. 1977). In addition, complexation with soil organic matter strongly reduces Cr(III) solubility (Reijonen 2017). As a consequence, Cr also showed low availability in our test soils, even in the absence of Fe.

The organic matter content of the soil (Feng et al. 2013) and its humic substances adsorbed onto kaolinite (Saada et al. 2003) had the greatest impact on arsenic adsorption. Balasoiu et al. (2001) pointed out that pH and oxidation reduction are the key chemical parameters influencing As sorption. The binding of As to soil is hardly affected by Ca (Kumpiene et al. 2008). In our study As showed rather high availability, which was the same for both the H_2_O and CaCl_2_ extractions, and can be explained by the presence of As as an anionic species. And the high availability might also be due to the low Fe content of the artificial soil, which prohibited the formation of As–Fe complexes. The fact that the type of organic matter (peat) used to prepare the artificial soil has a different structure compared to natural organic matter probably also contributed to the high As availability.

We found that As and Cr at a 30:70 Cu:metal ratio affected the Freundlich K_f_ for the sorption of Cu, with n values being <1 suggesting a concentration effect. K_f_ values for Cu were higher when based on H_2_O than 0.01 M CaCl_2_ extractable concentrations (Posthuma et al. 1997). The sorption of Cu was lower at the 10:90 Cr/Cu ratio and it increased at the high Cr/Cu ratios. The sorption of As in different Cr/Me ratios was quite similar, but n values indicated possible complexation of the metals (*n* > 1). Cr and As can form less soluble complexes (Kües 2007). As/Cr and Cr/As ratios were studied with *Staphylococcus xylosus* (Aryal et al. 2011) showing that As(V) ion at high concentration levels suppressed the sorption of Cr. Like in our case, As sorption increased with decreasing Cr level so increasing ratio of As/Cr. This is supported by Buchter et al. (1989) who observed greater retention for As compared to Cr.

### Toxicity of single metals

In our study, total soil concentrations up to 557 mg Cu/kg and 1224 mg Cr/kg did not affect the survival of *E. andrei*, but As did with an estimated LC_50_ of 92.5 mg/kg. The dose–response curves for the toxicity to earthworm reproduction of single Cr and Cu showed hormesis, which suggests a stimulus at low doses while high doses are toxic. For Cu, deficiency might have occurred in the control, explaining for the hormetic effect at the lower concentrations added to the artificial soil. Spurgeon et al. (2004) also found hormesis for Cu when testing growth and development of juveniles of *Lumbricus rubellus*. No indications for hormetic effects of Cr on earthworms were found in the literature. A hormetic effect of As was observed for juvenile reproduction of *E. fetida* at intermediate total concentrations (<45 mg/kg, dw) in low organic mining area soils (Neaman et al. 2012; Bustos et al. 2015). In our study, for As no signs of hormesis were seen, most likely because of the strong effects on earthworm reproduction already seen at the lowest concentrations tested.

Concentrations above 100 mg Cu/kg were toxic to the earthworms *Dendrodrilus rubidus* (Savigny) and *L. rubellus* in two different field-contaminated soils with 1.58% and 10.0% organic matter and pH (H_2_O) 7.18 and 5.14 (Langdon et al. 2001b). In OECD artificial soil, EC_50_ for the effect of Cu on the growth of *E. andrei* was >100 mg/kg (dw) (Van Gestel et al. 1991). Caetano et al. (2016) reported EC_50_ and EC_20_ values of 130.9 and 73 mg/kg (dw) for the effects of Cu on the reproduction of *E. andrei* in a Portuguese natural soil with pH (H_2_O) 5.9 and 6.5% OM. The EC_10_ and EC_50_ values for the toxicity of Cu to *E. fetida* in OECD artificial soil with pH (0.01 M CaCl_2_) 6.5 and 4.7% organic carbon were 225 and 349 mg/kg (dw), respectively (Criel et al. 2008). The EC_50_ and EC_10_ values of 154 and 74 mg Cu/kg dry soil, respectively found in our study are of the same order of magnitude as the values reported in the literature for different earthworm species.

LC_50_ values for the effects of Cr(III) and Cr(VI) on the survival of the earthworm *E. fetida* were 1656–1902 mg/kg and 222–257 mg/kg, respectively in field soil from India (Sivakumar and Subbhuraam 2005). Their soil contained only 0.14–0.68% organic carbon compared to about 5.8% in our artificial soil, explaining for the lower toxicity in our study and the absence of mortality at the highest test concentration (1500 mg/kg). Our EC_50_ and EC_10_ values for the effect of Cr on earthworm reproduction Cr were 449 mg/kg and 343 mg/kg. These values are in the same range as the 21-day EC_50_ for *E. fetida* (based on cocoon production) of 892 mg Cr/kg dw reported by Lock and Janssen (2002a).

EC_50_ and EC_10_ values for the effects of As on the cocoon production of *E. fetida* in an agricultural soil from Chile with pH (0.1 M KNO_3_) 5.7–7.6 and 0.7–5.8% OM were 22 and 8 mg/kg, respectively (Bustos et al. 2015). Lock and Janssen (2002b) reported an EC_50_ for effects on *E. fetida* cocoon production of 10.8 mg/kg (dw) in OECD artificial soil based on total concentration. EC_50_ values for the toxicity of As for *E. andrei* were 56–151 mg/kg (dw) and EC_10_ was 26 mg/kg (dw) when spiked as Na_2_HAsO_4_*7H_2_O into soils with an OM content <10.0% and pH-H_2_O 5.87–8.79 (Romero-Freire et al. 2015). The EC_50_ and EC_10_ obtained in our study (9.08 and 5.80 mg/kg dry soil, respectively) are in agreement with the ones reported by Bustos et al. (2015) and Lock and Janssen (2002b), but low compared to the study of Romero-Freire et al. (2015). We used the same As-salt as Romero-Freire et al. (2015) and our peat content was 10% and pH-H_2_O 6.27. Soil pH influences the uptake of the As by earthworms: At pH ≤ 6.75, internal As levels increased significantly (Peijnenburg et al. 1999a). The measured pH of our spiked OECD soils was <6.75. Together with the possible low Fe content of the artificial soil and the absence of ageing this may explain for the unexpected high As toxicity to *E. andrei* in our study.

### Mixture effects

Only the binary mixtures containing As showed negative, dose-related effects on the survival of *E. andrei* making it hard to interpret these mixture effects. For reproduction, antagonistic effects were found for the all binary mixtures of Cu–As, Cu–Cr and Cr–As when tested against the CA model and based on total measured soil concentrations. Using cocoon production of *E. fetida*, Spurgeon and Hopkin (1995) found antagonistic effects for mixtures of Cu, Zn, Cd and Pb. Khalil et al. (1996) reported that mixtures of Cu, Cd and Zn were antagonistic to the cocoon production of the earthworm *A. caliginosa*, also using the CA model.

When relating mixture effects to H_2_O and 0.01 M CaCl_2_ extractable concentrations, similar antagonistic effects in binary mixtures of Cu–As, Cu–Cr and Cr–As on the reproduction of *E. andrei* were seen against the CA model. Dose-ratio dependent deviation was detected for H_2_O and 0.01 M CaCl_2_ extractable concentrations at low Cu switching to synergism in the Cu–Cr mixture and for the Cr–As mixture related to total concentrations at low Cr concentrations for both reference models of CA and IA. Related to the IA model, mainly additivity was seen when expressing effects on the basis of total and H_2_O or 0.01 M CaCl_2_ extractable concentrations.

Van Gestel and Hensbergen (1997) reported that interactions between Cd and Zn were antagonistic which was explained from interactions at the level of sorption to the soil, so from K_f_ values. This seems confirmed by the K_f_ values obtained in our study showing a stronger sorption when the other element was present (Table S1–S3 in the Supporting Information). This explains antagonism when relating effects to total concentrations and a shift to additivity when effects were related to H_2_O and 0.01 M CaCl_2_ extractable concentrations (Table 6).

The results of this study suggest that Cu, Cr and As have different modes of action as they generally showed antagonism when their mixture effect was analyzed using the CA model and additivity according to the IA model. The differences in uptake and elimination kinetics found in our earlier study (Kilpi-Koski et al. 2019) may have contributed to the rather high toxicity of As in this study and to the dissimilar action of the three metals in the mixtures.

## Conclusion

In the present study we found that arsenic (As) was the most toxic metal to earthworms. Cr and Cu showed hormesis at low concentrations. Extractable concentrations showed that As had a high availability in the OECD artificial soil, which was confirmed by its high toxicity. Although availability was fairly high and did not differ between the H_2_0 and 0.01 M CaCl_2_ extractions, the mixtures of As, Cr and Cu generally showed antagonism against the CA model for their toxicity to the reproduction of *Eisenia andrei*. This may be explained from complexation reactions in the soil but also from a different mode of action of the three metals used in chromated copper arsenate (CCA) mixtures used for wood preservation. Our results showed that CCA components interact with each other leading to a reduced toxicity in the mixtures.

## Supplementary information

Supporting Information
