# Supplementary material for: Toxicity of binary mixtures of Cu, Cr and As to the earthworm Eisenia andrei
Source: Ecotoxicology. 2020 Jun 25;29(7):900–11. doi: 10.1007/s10646-020-02240-1 (PMC7427711; doi:10.1007/s10646-020-02240-1)
Supplement: Supplementary file 1 — Supporting Information [file 10646_2020_2240_MOESM1_ESM.docx]

**Supporting Information to**

“Toxicity of binary mixtures of Cu, Cr and As to the earthworm *Eisenia andrei* “

Johanna Kilpi-Koski*, Olli-Pekka Penttinen*, Ari O. Väisänen^#^ and Cornelis A.M. van Gestel^§^

*Department of Environmental Sciences, Faculty of Biological and Environmental Sciences, University of Helsinki, Niemenkatu 73, 15140 Lahti, Finland

^#^Department of Chemistry, University of Jyväskylä, PL 35, 40014 Jyväskylän yliopisto, Jyväskylä, Finland

^§^Department of Ecological Science, Faculty of Science, Vrije Universiteit, De Boelelaan 1085, 1081 HV, Amsterdam, The Netherlands

Fig S1. Nominal concentrations of the individual metals and the binary mixtures of Cu-Cr (top), Cu-As (middle) and As-Cr (bottom) based on expected toxic strengths of 0.25, 0.5, 1, 2 and 4 TU for Cu and 0.16, 0.4, 1, 2.5 and 6.25 for Cr and As. The mixtures tested had toxicant ratios of 1:1, 9:1, 1:9, 1:3 and 3:1.

Fig S2. Comparison of Freundlich K_f_ values for the partitioning of Cu in the binary mixture toxicity tests with As and Cr, based on 0.01 M CaCl_2_ (left) and H_2_0-extractable concentrations (right) in OECD artificial soil. The error bars show Standard Errors (SE). For both the mixtures with As and Cr, the sorption of Cu is a bit less at a low Cu-As and Cu-Cr ratios but higher at the higher Cu/metal ratios.

Fig S3. Freundlich K_f_ values for the partitioning of Cr in the binary mixture toxicity tests with As and Cu, based on 0.01 M CaCl_2_ and H_2_0-extractable concentrations in OECD artificial soil. The error bars show Standard Errors (SE). A strange outlier was seen at Cr-Cu 50:50, but overall trend is a lower sorption at the 10:90 Cr/Cu ratio (suggesting an effect specific for Cu/Cr) and an increased sorption at the high Cr/Me ratios.

Fig S4. Freundlich K_f_ values for the partitioning of As in the binary mixture toxicity tests with Cr and Cu, based on 0.01 M CaCl_2_ and H_2_0-extractable concentrations in OECD artificial soil. The error bars show Standard Errors (SE). Interactions are scattered, with a stronger sorption at the 10:90 As/Me ratio but a low sorption at the 30:70 As/Cr ratio that increased again with increasing ratio of As/Cr. Such trend was not visible for Cu.

Fig S5. Hormetic dose-response curves for the effect of single Cu and Cr exposure on the reproduction of *Eisenia andrei* in OECD artificial soil. The R^2^ values showing the goodness of fit of the hormetic model were 0.874 and 0.867 for Cu and Cr, respectively.

Table S1. Freundlich K_f_ and n values (± SE) for the partitioning of Cu in the binary mixture toxicity tests with As and Cr, based on 0.01 M CaCl_2_ and H_2_0-extractable concentrations in OECD artificial soil.

|  | **K_f_ (L/kg)^n^** | **n** |  | **Kf (L/kg)^n^** | **n** |
| --- | --- | --- | --- | --- | --- |
| **Cu single CaCl_2_** | 91.4 ± 1.11 | 0.57 ± 0.07 | **Cu single CaCl_2_** | 91.4 ± 1.11 | 0.57 ± 0.07 |
| **Cu - As 10:90 CaCl_2_** | 75.3 ± 1.54 | 0.59 ± 0.18 | **Cu - Cr 10:90 CaCl_2_** | 56.0 ± 1.22 | 0.41 ± 0.07 |
| **Cu - As 30:70 CaCl_2_** | 99.8 ± 1.09 | 0.62 ± 0.07 | **Cu - Cr 30:70 CaCl_2_** | 120 ± 1.11 | 0.73 ± 0.08 |
| **Cu - As 50:50 CaCl_2_** | 109 ± 1.05 | 0.79 ± 0.05 | **Cu - Cr 50:50 CaCl_2_** | 117 ± 1.00 | 0.80 ± 0.04 |
| **Cu - As 70:30 CaCl_2_** | 113 ± 1.05 | 0.75 ± 0.06 | **Cu - Cr 70:30 CaCl_2_** | 126 ± 1.12 | 0.64 ± 0.15 |
| **Cu - As 90:10 CaCl_2_** | 106 ± 1.03 | 0.70 ± 0.04 | **Cu - Cr 90:10 CaCl_2_** | 116 ± 1.05 | 0.67 ± 0.05 |
| **Cu single H_2_O** | 82.9 ± 1.11 | 0.58 ± 0.06 | **Cu single H_2_O** | 82.9 ± 1.11 | 0.58 ± 0.06 |
| **Cu - As 10:90 H_2_O** | 84.3 ± 1.44 | 0.72 ± 0.17 | **Cu - Cr 10:90 H_2_O** | 81.8 ± 1.39 | 0.76 ± 0.17 |
| **Cu - As 30:70 H_2_O** | 93.4 ± 1.05 | 0.70 ± 0.04 | **Cu - Cr 30:70 H_2_O** | 105 ± 1.09 | 0.80 ± 0.08 |
| **Cu - As 50:50 H_2_O** | 93.5 ± 1.05 | 0.83 ± 0.04 | **Cu - Cr 50:50 H_2_O** | 98.8 ± 1.03 | 0.85 ± 0.03 |
| **Cu - As 70:30 H_2_O** | 99.8 ± 1.05 | 0.82 ± 0.06 | **Cu - Cr 70:30 H_2_O** | 111 ± 1.12 | 0.69 ± 0.16 |
| **Cu - As 90:10 H_2_O** | 95.4 ± 1.04 | 0.74 ± 0.04 | **Cu - Cr 90:10 H_2_O** | 105 ± 1.06 | 0.70 ± 0.06 |

Table S2. Freundlich K_f_ and n values (± SE) for the partitioning of Cr in the binary mixture toxicity tests with Cu and As, based on 0.01 M CaCl_2_ and H_2_0-extractable concentrations in OECD artificial soil.

|  | **K_f_ (L/kg)^n^** | **n** |  | **K_f_ (L/kg)^n^** | **n** |
| --- | --- | --- | --- | --- | --- |
| **Cr single CaCl_2_** | 62.4 ± 1.10 | 1.23 ± 0.07 | **Cr single CaCl_2_** | 62.4 ± 1.10 | 1.23 ± 0.07 |
| **Cr - Cu 10:90 CaCl_2_** | 43.4 ± 1.13 | 1.01 ± 0.11 | **Cr - As 10:90 CaCl_2_** | 63.4 ± 1.24 | 1.21 ± 0.18 |
| **Cr - Cu 30:70 CaCl_2_** | 62.4 ± 1.14 | 0.91 ± 0,19 | **Cr - As 30:70 CaCl_2_** | 62.4 ± 1.05 | 1.08 ± 0.07 |
| **Cr - Cu 50:50 CaCl_2_** | 9.13 | 0.097 | **Cr - As 50:50 CaCl_2_** | 58.2 ± 1.07 | 1.00 ± 0.06 |
| **Cr - Cu 70:30 CaCl_2_** | 72.0 ± 1.09 | 1.03 ± 0.09 | **Cr - As 70:30 CaCl_2_** | 66.7 ± 1.11 | 1.08 ± 0.12 |
| **Cr - Cu 90:10 CaCl_2_** | 82.9 ± 1.12 | 1.02 ± 0.10 | **Cr - As 90:10 CaCl_2_** | 63.7 ± 1.71 | 1.47 ± 0.49 |
| **Cr single H_2_O** | 49.1 ± 1.10 | 1.34 ± 0.05 | **Cr single H_2_O** | 49.1 ± 1.10 | 1.34 ± 0.05 |
| **Cr - Cu 10:90 H_2_O** | 38.8 ± 1.12 | 1.06 ± 0.12 | **Cr - As 10:90 H_2_O** | 56.8 ± 1.16 | 1.23 ± 0.14 |
| **Cr - Cu 30:70 H_2_O** | 54.6 ± 1.14 | 0.92 ± 0.19 | **Cr - As 30:70 H_2_O** | 54.5 ± 1.05 | 1.07 ± 0.08 |
| **Cr - Cu 50:50 H_2_O** | 49.5 ± 1.05 | 1.19 ± 0.04 | **Cr - As 50:50 H_2_O** | 51.0 ± 1.08 | 1.10 ± 0.06 |
| **Cr - Cu 70:30 H_2_O** | 59.8 ± 1.10 | 1.09 ± 0.09 | **Cr - As 70:30 H_2_O** | 58.3 ± 1.22 | 1.07 ± 0.12 |
| **Cr - Cu 90:10 H_2_O** | 60.0 ± 1.14 | 1.13 ± 0.11 | **Cr - As 90:10 H_2_O** | 59.1 ± 1.80 | 1.37 ± 0.48 |

Table S3. Freundlich K_f_ and n values (± SE) for the partitioning of As in the binary mixture toxicity tests with Cr and Cu, based on 0.01 M CaCl_2_ and H_2_0-extractable concentrations in OECD artificial soil.

|  | **K_f_ (L/kg)** | **SE** |  | **K_f_ (L/kg)** | **n** |
| --- | --- | --- | --- | --- | --- |
| **As single CaCl_2_** | 5.96 ± 1.12 | 1.12 | **As single CaCl_2_** | 5.96 ± 1.12 | 0.98 ± 0.04 |
| **As - Cr 10:90 CaCl_2_** | 9.65 ± 1.26 | 1.26 | **As - Cu 10:90 CaCl_2_** | 10.9 ± 1.76 | 1.14 ± 0.72 |
| **As - Cr 30:70 CaCl_2_** | 3.98 ± 1.30 | 1.30 | **As Cu 30:70 CaCl_2_** | 7.17 ± 1.18 | 0.86 ± 0.09 |
| **As - Cr 50:50 CaCl_2_** | 6.31 ± 1.17 | 1.17 | **As - Cu 50:50 CaCl_2_** | 7.51 ± 1.23 | 0.90 ± 0.08 |
| **As - Cr 70:30 CaCl_2_** | 7.49 ± 1.30 | 1.30 | **As - Cu 70:30 CaCl_2_** | 7.40± 1.22 | 0.93 ± 0.08 |
| **As - Cr 90:10 CaCl_2_** | 7.51 ± 1.17 | 1.17 | **As - Cu 90:10, CaCl_2_** | 5.28 ± 1.47 | 1.05 ± 0.14 |
| **As single H_2_O** | 5.86 ± 1.16 | 1.16 | **As single H_2_O** | 5.86 ± 1.16 | 0.98 ± 0.05 |
| **As - Cr 10:90 H_2_O** | 10.3 ± 1.21 | 1.21 | **As - Cu 10:90 H_2_O** | 10.7 ± 1.84 | 0.99 ± 0.68 |
| **As - Cr 30:70 H_2_O** | 4.09 ± 1.30 | 1.30 | **As - Cu 30:70 H_2_O** | 7.55 ± 1.21 | 0.87 ± 0.11 |
| **As - Cr 50:50 H_2_O** | 5.64 ± 1.23 | 1.23 | **As -Cu 50:50 H_2_O** | 7.95 ± 1.22 | 0.91 ± 0.08 |
| **As - Cr 70:30 H_2_O** | 6.23 ± 1.30 | 1.30 | **As - Cu 70:30 H_2_O** | 7.48 ± 1.14 | 0.93 ± 0.05 |
| **As - Cr 90:10 H_2_O** | 7.41 ± 1.18 | 1.18 | **As - Cu 90:10 H_2_O** | 8.96 ± 1.34 | 0.84 ± 0.11 |

Table S4. Effects of a binary mixture of the **copper (Cu) – arsenic (As)** on the reproduction of *Eisenia andrei* (number of juveniles per pot) exposed in spiked OECD artificial soil, based on H_2_O extractable concentrations. Values are calculated using **the concentration addition model (CA)** and **the** **independent action model (IA)** extended with deviation parameter *a* to show synergism/antagonism (S/A) and an additional deviation parameter *b* for dose ratio-dependent deviation (DR) or dose level-dependent deviation (DL). The *p(X^2^)* values indicate the significance of the additional deviation parameters.

|  | **H_2_O extract** | | | | | | | |
| --- | --- | --- | --- | --- | --- | --- | --- | --- |
|  | **CA** | **IA** | **S/A** | | **DR** | | **DL** | |
|  |  |  | **CA** | **IA** | **CA** | **IA** | **CA** | **IA** |
| **Max** | 39.1 | 40.4 | 39.3 | 40.8 | 39.6 | 39.5 | 44.6 | 40.0 |
| **Slope Cu** | 2.36 | 2.11 | 2.24 | 1.98 | 2.64 | 3.39 | 0.645 | 1.13 |
| **Slope As** | 4.74 | 3.25 | 4.42 | 3.07 | 4.02 | 4.30 | 1.51 | 2.57 |
| **EC_50_ Cu (mg/kg)** | 11.0 | 8.88 | 9.86 | 8.17 | 19.4 | 24.1 | 3.07 | 5.02 |
| **EC_50_ As (mg/kg)** | 8.32 | 6.80 | 7.70 | 6.46 | 7.42 | 7.66 | 2.80 | 5.85 |
| **a** |  |  | 0.666 | 0.631 | 1.11 | -1.91 | **18.0** | 9.72 |
| **b** |  |  |  |  | -5.44 | -10.3 | **0.192** | 1.01 |
| **chi = p(x^2^)** |  |  | 0.292 | 0.513 | 0.147 | 0.230 | **0.016** | 0.539 |
| **R^2^** | 0.890 | 0.894 | 0.891 | 0.895 | 0.894 | 0.896 | 0.899 | 0.895 |

When testing the Cu-As mixture against CA and IA models based on H_2_O extractable concentrations, we found no deviations from CA, but DL dependent deviation from S/A from the CA model. There are deviations under DL model for CA indicating antagonism. At high concentrations above the EC_50_ a shift occurs from antagonism to synergism. No significant deviations from IA indicating that Cu and As have dissimilar modes of action.

Table S5. Effects of a binary mixture of the **copper (Cu) – chromium (Cr)** on the reproduction of *Eisenia andrei* (number of juveniles per pot) exposed in spiked OECD artificial soil, based on **H_2_O extractable concentrations.** Values are calculated using **the concentration addition model (CA)** and **the** **on independent action model (IA)** extended with deviation parameter *a* to show synergism/antagonism (S/A) and an additional deviation parameter *b* for dose ratio-dependent deviation (DR) or dose level-dependent deviation (DL). The *p(X^2^)* values indicate the significance of the additional deviation parameters.

|  | **H_2_O extract** | | | | | | | | |
| --- | --- | --- | --- | --- | --- | --- | --- | --- | --- |
|  | **CA** | **IA** | **S/A** | | **DR** | | **DL** | |  |
|  |  |  | **CA** | **IA** | **CA** | **IA** | **CA** | **IA** |  |
| **Max** | 41.4 | 40.6 | 40.8 | 40.6 | 40.0 | 40.5 | 40.9 | 41.0 |  |
| **Slope Cu** | 1.82 | 1.97 | 1.85 | 1.97 | 1.92 | 2.06 | 1.98 | 2.11 |  |
| **Slope Cr** | 2.57 | 2.50 | 2.87 | 2.41 | 2.92 | 2.03 | 3.06 | 2.40 |  |
| **EC_50_ Cu (mg/kg)** | 7.94 | 6.97 | 6.46 | 6.78 | 8.61 | 8.86 | 6.42 | 6.12 |  |
| **EC_50_ Cr (mg/kg)** | 33.7 | 31.9 | 30.1 | 31.4 | 25.9 | 25.6 | 30.2 | 29.6 |  |
| **a** |  |  | **1.66** | 0.302 | **7.09** | **7.59** | 1.00 | 0.0014 |  |
| **b** |  |  |  |  | **-11.1** | **-14.3** | -0.420 | -1570 |  |
| **chi = p(x^2^)** |  |  | **0.015** | 0.658 | **0.012** | **0.015** | no number | 0.314 |  |
| **R^2^** | 0.752 | 0.764 | 0.767 | 0.764 | 0.782 | 0.778 | 0.761 | 0.767 |  |

For the Cu – Cr mixture there is significant antagonism with a significant dose ratio dependence (DR) deviation when tested against the CA model. The additional parameters of DR were a = 7.09 and b = -11.1. The IA model showed no deviation, although a significant DR dependent deviation was seen (a = 7.59 and b = -14.3). Both the CA and IA models indicate that Cu and Cr have similar modes of action.

Table S6. Effects of a binary mixture of the **chromium (Cr) – As (arsenic)** on the reproduction of *Eisenia andrei* (number of juveniles per pot) exposed in spiked OECD artificial soil, based on H_2_O extractable concentrations. Values are calculated using **the concentration addition model (CA)** and **the** **on independent action model (IA)** extended with deviation parameter *a* to show synergism/antagonism (S/A) and an additional deviation parameter *b* for dose ratio-dependent deviation (DR) or dose level-dependent deviation (DL). The *p(X^2^)* values indicate the significance of the additional deviation parameters.

|  | **H_2_O extract** | | | | | | | |
| --- | --- | --- | --- | --- | --- | --- | --- | --- |
|  | **CA** | **IA** | **S/A** | | **DR** | | **DL** | |
|  |  |  | **CA** | **IA** | **CA** | **IA** | **CA** | **IA** |
| **Max** | 42.0 | 39.9 | 47,9 | 48.6 | 48.2 | 47.0 | 47.7 | 48.7 |
| **Slope Cr** | 4.67 | 52.6 | 282000 | 1.70 | 22500 | 1.84 | 62800 | 1.73 |
| **Slope As** | 2.26 | 2.63 | 1.07 | 1.12 | 1.07 | 1.10 | 1.05 | 1.14 |
| **EC_50_ Cr (mg/kg)** | 35.1 | 27.7 | 8.21 | 6.19 | 8.80 | 5.13 | 7.98 | 6.39 |
| **EC_50_ As (mg/kg)** | 6.51 | 6.27 | 1.44 | 1.04 | 1.40 | 1.32 | 1.41 | 1.07 |
| **a** |  |  | **9.24** | **9.99** | 10.2 | 4.29 | 9.82 | 8.70 |
| **b** |  |  |  |  | -2.30 | 12.4 | 0.00764 | -0.14 |
| **chi = p(x^2^)** |  |  | **4.0*10^-24^** | **2.8*10^-12^** | 0.814 | 0.098 | 0.708 | 0.741 |
| **R^2^** | 0.863 | 0.911 | 0.951 | 0.946 | 0.951 | 0.947 | 0.951 | 0.946 |

For the Cr-As binary mixture the CA model points at antagonism, with no further DR and DL deviations. The MIXTOX model estimated an extremely steep slope for Cr and a fairly flat one for As, while also the EC_50_ values for both metals were rather low compared to the single exposures. The steep slope may be due to the As data. Rerunning the model with the slope for Cr fixed at a value of 5 gave a similar conclusion: antagonism. The IA model also showed a significant antagonism and no DR or DL deviations. So, both models indicate that Cr and As have dissimilar modes of action.

Table S7. Effects of a binary mixture of the **copper (Cu) – As (arsenic)** on the reproduction of *Eisenia andrei* (number of juveniles per pot) exposed in spiked OECD artificial soil, based on CaCl_2_ extractable concentrations. Values are calculated using **the concentration addition model (CA)** and **the** **on independent action model (IA)** extended with deviation parameter *a* to show synergism/antagonism (S/A) and an additional deviation parameter *b* for dose ratio-dependent deviation (DR) or dose level-dependent deviation (DL). The *p(X^2^)* values indicate the significance of the additional deviation parameters.

|  | **CaCl_2_ extract** | | | | | | | |
| --- | --- | --- | --- | --- | --- | --- | --- | --- |
|  | **CA** | **IA** | **S/A** | | **DR** | | **DL** | |
|  |  |  | **CA** | **IA** | **CA** | **IA** | **CA** | **IA** |
| **Max** | 38,6 | 38.7 | 38.6 | 38.9 | 38.7 | 38.7 | 38.6 | 38.7 |
| **Slope Cu** | 2.33 | 2.12 | 2.25 | 4.02 | 2.34 | 3.49 | 2.28 | 1.18 |
| **Slope As** | 6.76 | 5.81 | 6.82 | 6.76 | 6.14 | 5.93 | 6.87 | 4.39 |
| **EC_50_ Cu (mg/kg)** | 10.3 | 8.17 | 9.49 | 24.5 | 15.3 | 22.5 | 9.54 | 5.17 |
| **EC_50_ As (mg/kg)** | 8.30 | 7.24 | 7.95 | 8.26 | 7.61 | 8.03 | 7.97 | 6.90 |
| **a** |  |  | 0.47 | **-9.94** | 0.99 | -5.91 | 0.377 | 7.59 |
| **b** |  |  |  |  | -3.79 | -4.16 | -0.161 | 1.13 |
| **chi = p(x^2^)** |  |  | 0.655 | **0.028** | 0.173 | 0.414 | 0.950 | n.s.* |
| **R^2^** | 0.900 | 0.900 | 0.900 | 0.905 | 0.902 | 0.905 | 0.900 | 0.902 |

*very poor fit, did not even provide p value.

Based on CaCl_2_ extractable concentrations the CA model did not show any significant deviations from additivity. The IA model did show a significant deviation from additivity, with a = -9.94 indicating synergism. This suggests that Cu and As have similar modes of action when toxicity is expressed on the basis of CaCl_2_ extractable soil concentrations.

Table S8. Effects of a binary mixture of the **copper (Cu) - chromium (Cr)** on the reproduction of *Eisenia andrei* (number of juveniles per pot) exposed for 8 weeks in spiked OECD artificial soil, based on CaCl_2_ extractable concentrations. Values are calculated using **the concentration addition model (CA)** and **the** **on independent action model (IA)** extended with deviation parameter *a* to show synergism/antagonism (S/A) and an additional deviation parameter *b* for dose ratio-dependent deviation (DR) or dose level-dependent deviation (DL). The *p(X^2^)* values indicate the significance of the additional deviation parameters.

|  | **CaCl_2_ extract** | | | | | | | |
| --- | --- | --- | --- | --- | --- | --- | --- | --- |
|  | **CA** | **IA** | **S/A** | | **DR** | | **DL** | |
|  |  |  | **CA** | **IA** | **CA** | **IA** | **CA** | **IA** |
| **Max** | 40.8 | 41.8 | 41.2 | 41.2 | 41.1 | 41.3 | 41.1 | 42.0 |
| **Slope Cu** | 1.80 | 1.60 | 1.78 | 1.72 | 1.79 | 1.95 | 1.99 | 2.15 |
| **Slope Cr** | 2.12 | 1.58 | 2.08 | 2.22 | 1.93 | 1.64 | 2.31 | 2.66 |
| **EC_50_ Cu (mg/kg)** | 6.78 | 6.05 | 7.77 | 8.04 | 7.71 | 7.83 | 7.75 | 7.25 |
| **EC_50_ Cr (mg/kg)** | 28.2 | 26.1 | 30.1 | 30.8 | 28.2 | 28.4 | 30.1 | 29.2 |
| **a** |  |  | -0.882 | **-2.43** | 1.80 | 1.31 | -1.68 | -4.53 |
| **b** |  |  |  |  | -4.75 | -5.88 | 0.481 | 1.17 |
| **chi = p(x^2^)** |  |  | 0.281 | **0.047** | 0.156 | 0.210 | 0.455 | 0.122 |
| **R^2^** | 0.674 | 0.665 | 0.677 | 0.678 | 0.684 | 0.683 | 0.679 | 0.686 |

Testing the Cu-Cr binary mixture based on CaCl2-extractable concentrations no deviation against the CA model was seen. In the IA model there was slight but significant synergism with a = 2.43 for S/A, but no DR or DL deviations. Cu and Cr have similar modes action based on the CA and IA models.

Table S9. Effects of a binary mixture of the **chromium (Cr) – As (arsenic)** on the reproduction of *Eisenia andrei* (number of juveniles per pot) exposed for 8 weeks in spiked OECD artificial soil, based on CaCl_2_ extractable concentrations. Values are calculated using **the concentration addition model (CA)** and **the** **on independent action model (IA)** extended with deviation parameter *a* to show synergism/antagonism (S/A) and an additional deviation parameter *b* for dose ratio-dependent deviation (DR) or dose level-dependent deviation (DL). The *p(X^2^)* values indicate the significance of the additional deviation parameters.

|  | **CaCl_2_ extract** | | | | | | | |
| --- | --- | --- | --- | --- | --- | --- | --- | --- |
|  | **CA** | **IA** | **S/A** | | **DR** | | **DL** | |
|  |  |  | **CA** | **IA** | **CA** | **IA** | **CA** | **IA** |
| **Max** | 42.1 | 40.2 | 53.0 | 51.3 | 53.2 | 50.0 | 52.9 | 51.8 |
| **Slope Cr** | 3.18 | 3.72 | 1.50 | 1.29 | 1.32 | 1.34 | 1.49 | 1.32 |
| **Slope As** | 2.27 | 3.04 | 1.41 | 1.36 | 1.39 | 1.30 | 1.40 | 1.38 |
| **EC_50_ Cr (mg/kg)** | 34.3 | 26.3 | 6.28 | 5.72 | 4.63 | 4.44 | 6.27 | 5.79 |
| **EC_50_ As (mg/kg)** | 6.88 | 6.80 | 1.62 | 1.56 | 1.76 | 1.93 | 1.62 | 1.57 |
| **a** |  |  | **7.73** | **7.89** | 3.29 | 2.27 | 7.80 | 6.70 |
| **b** |  |  |  |  | 9.55 | 11.2 | 0.001 | -0.19 |
| **chi = p(x^2^)** |  |  | **5.1*10^-18^** | **2.4*10^-13^** | 0.438 | 0.09 | 0.952 | 0.736 |
| **R^2^** | 0.848 | 0.877 | 0.928 | 0.928 | 0.928 | 0.930 | 0.928 | 0.928 |

Based on both the CA and IA models there was highly significant antagonism (a>0), but no further DR or DL deviations.
